# Antioxidant, Antibacterial, Cytotoxic, and Anti-Inflammatory Potential of the Leaves of *Solanum lycocarpum* A. St. Hil. (Solanaceae)

**DOI:** 10.1155/2015/315987

**Published:** 2015-05-04

**Authors:** Guilherme Augusto Ferreira da Costa, Melissa Grazielle Morais, Aline Aparecida Saldanha, Izabela Caputo Assis Silva, Álan Alex Aleixo, Jaqueline Maria Siqueira Ferreira, Adriana Cristina Soares, Joaquim Maurício Duarte-Almeida, Luciana Alves Rodrigues dos Santos Lima

**Affiliations:** Universidade Federal de São João Del-Rei, Campus Centro-Oeste Dona Lindu, 35501-296 Divinópolis, MG, Brazil

## Abstract

Ethanol extract and fractions obtained from leaves of *Solanum lycocarpum* were examined in order to determine their phenolic composition, antioxidant, antibacterial, anti-inflammatory, and cytotoxic potential. High performance liquid chromatography coupled with DAD analysis indicated that the flavonoids apigenin and kaempferol were the main phenolic compounds present in dichloromethane and ethyl acetate fractions, respectively. The antioxidant activity was significantly more pronounced for dichloromethane, ethyl acetate, and hydroethanol fractions than that of the commercial antioxidant 2,6-di-tert-butyl-4-methylphenol. The hexane and dichloromethane fractions were more active against the tested bacteria. The hydroethanol fraction exhibited significant anti-inflammatory activity at the dose of 75 and 150 mg/kg in the later phase of inflammation. However, the antiedematogenic effect of the higher dose of the ethyl acetate fraction (150 mg/kg) was more pronounced. The ethyl acetate fraction also presented a less cytotoxic effect than the ethanol extract and other fractions. These activities found in *S. lycocarpum* leaves can be attributed, at least in part, to the presence of phenolic constituents such as flavonoids. This work provided the knowledge of phenolic composition in the extract and fractions and the antioxidant, antibacterial, anti-inflammatory, and cytotoxic activities of leaves of *S. lycocarpum*.

## 1. Introduction

Polyphenolic compounds, such as flavonoids and phenolic acids, are commonly consumed as natural components of vegetables, beans, fruits, and/or phytotherapeutics and are generally regarded as safe chemicals, thus displaying low toxicological activity [1]. These compounds have been presented as therapeutic alternatives for the treatment of a large number of pathologies [2]. Flavonoids, particularly apigenin and luteolin, have shown potent activity against oxidative stress [3]. Other studies in several systems have revealed that apigenin was able to inhibit tumor growth [4]. Additionally, recent studies have shown anti-inflammatory, antimicrobial, and antioxidant properties for kaempferol and kaempferol rhamnosides [5, 6]. Cinnamic acids, such as caffeic and chlorogenic acids, are also very well known for their antioxidant properties [7, 8].

The Solanaceae family comprises about 3000 species and 150 genera. It is prevalent in tropical and subtropical regions of South America and has economic importance because several species of the* Solanum* genus are cultivated for food, such as* Solanum tuberosum* (potato),* Solanum lycopersicum* (tomato),* Solanum melongena* (eggplant), and* Solanum gilo* (gilo) [9]. The species* Solanum lycocarpum* A. St. Hil., popularly known as the “fruit of the wolf,” is widely distributed in the Brazilian Cerrado. The fruits are usually consumed “in natura” or used in jellies, jams, or pasta preparations [10]. It is widely used in traditional medicine as a sedative, in the treatment of epilepsy, asthma, diabetes, obesity, the reduction of cholesterol levels, and abdominal and renal pains [11]. Biological activities such as anti-inflammatory, antioxidant, cytotoxic, antibacterial, allelopathic, and larvicidal have been reported for this species [10, 12–14].

Phytochemical studies of this species showed that solasonine and solamargine are two predominant steroidal glycoalkaloids, which have a common aglycone, solasodine [15]. A recent study also reported the presence of cinnamic acids, such as caffeic and chlorogenic acids, in ripe fruits of* S. lycocarpum* [13]. However, until now, no data exist about the phenolic composition of the leaves of* S. lycocarpum* and their antioxidant, antibacterial, anti-inflammatory, and cytotoxic activities. The aim of this study was to identify the main phenolic compounds and estimate the total flavonoid content of the ethanol extract (EE) and fractions obtained from the leaves of* S. lycocarpum* and also to demonstrate their antioxidant, antibacterial, anti-inflammatory, and cytotoxic potential.

## 2. Materials and Methods

### 2.1. Chemicals

Ascorbic acid (AA), 2,6-di-tert-butyl-4-methylphenol (BHT), 1,1-diphenyl-2-picrylhydrazyl (DPPH), kaempferol, quercetin, rutin, apigenin, luteolin, coumaric acid, 3-(4,5-dimethylthiazol-2-yl)-2,5-diphenyltetrazolium bromide (MTT), carrageenan *λ* type IV, indomethacin, HPLC grade methanol, and acetonitrile were obtained from Sigma (St. Louis, MO, USA). Caffeic, ferulic, and chlorogenic acids were purchased from Apin Chemicals Ltd. (Abingdon, UK). Mueller Hinton broth (MH) medium was acquired from Himedia (Mumbai, India). Streptomycin, penicillin, and fetal bovine serum (FBS) were purchased from Cultilab (Campinas, SP, Brazil). Dulbecco's Modified Eagle's Medium (DMEM) was purchased from Gibco (Grand Island, NY, USA). All other reagents were of analytical grade and were obtained from Vetec (Duque de Caxias, RJ, Brazil).

### 2.2. Plant Material and Extraction

The leaves of* S. lycocarpum* A. St. Hil. were collected in São Sebastião do Oeste, Minas Gerais, Brazil, in August 2011. The plant material was identified by Dr. Alexandre Salino and a voucher specimen (BHCB 159397) was deposited at the Instituto de Ciências Biológicas Herbarium, Universidade Federal de Minas Gerais, Belo Horizonte, MG, Brazil. As* S. lycocarpum* a Brazilian native genetic material, the present study had been approved by the Conselho Nacional de Desenvolvimento Científico e Tecnológico (CNPq) through Access and Shipment Component of Genetic Heritage for scientific research purpose (number 010655/2011-5).

Extraction of the dried and powdered leaves (121.00 g) by percolation (EtOH, 7 L, 72 h) gave the EE (17.02 g). Part of this extract (7.90 g) was dissolved in EtOH/H_2_O (1 : 1) and successively extracted with C_6_H_14_, CH_2_Cl_2_, and EtOAc, resulting in 1.90, 0.48, 0.67, and 3.20 g of hexane (Hex), dichloromethane (DCM), ethyl acetate (Ac), and hydroethanol (HE) fractions.

### 2.3. Total Flavonoid Content

The total flavonoid contents were estimated according to the Dowd method [16], with modifications. Exactly 2 mL of 2% aluminum trichloride (AlCl_3_) in methanol was mixed with the same volume of the extract or fraction solution (1.0 mg/mL). The absorbance was read at 415 nm using a Hitachi 2010 spectrophotometer after 10 min, with a blank sample consisting of a 2 mL extract or fraction solution with 2 mL methanol without AlCl_3_. Rutin was used as the reference compound to produce the standard curve, and total flavonoid contents were expressed as *μ*g of rutin equivalents/mg of extract or fraction. All assays were performed in triplicate.

### 2.4. Phenolic Profiles by HPLC-DAD

The phenolic substances in the ethanol extract and fractions were carried out using analytical reversed phase HPLC on an Agilent 1260 system with an autosampler and quaternary pump coupled with a diode array detector. Compound separation was performed by a Zorbax Eclipse Plus 5B RP-18 (5 *μ*m, 250 × 4.6 mm, Agilent, USA) column. The mobile phases were (A) water/formic acid (99.9 : 0.1) and (B) acetonitrile. The gradient consisted of 20% of (B) for 2 minutes and increased to 30% of (B) after 10 minutes, 50% of (B) after another 10 minutes, and 70% of (B) after an additional 10 minutes. For column cleaning, 90% of phase (B) was used [7]. Each sample was injected in duplicate, ranging from 5 to 20 *μ*L according to the sample concentration. Determination was performed by comparing the retention times and the UV spectra against those obtained from the standards (apigenin, kaempferol, luteolin, quercetin, rutin, caffeic, coumaric, ferulic, and chlorogenic acids). The determination was positive when the similarity between the chromatograms was equal to or greater than 90%.

### 2.5. DPPH Radical Scavenging Assay

The radical scavenging abilities of extract and fractions of* S. lycocarpum* were based on reactions with 1,1-diphenyl-2-picrylhydrazyl radical (DPPH) and compared to standards, 2,6-di-tert-butyl-4-methylphenol (BHT) and ascorbic acid (AA). Determination of antioxidant activity by the DPPH method was adapted for use with microplates [17]. Briefly, a solution of DPPH (0.002% w/v) was prepared in 80% methanol. Volumes of 75 *μ*L of samples or standards (1, 10, 100, 250, and 500 *μ*g/mL) were added to wells in a 96-well flat-bottom plate containing 150 *μ*L of DPPH solution. The plate was then covered and left in the dark at room temperature (25°C). After 30 min, absorbance at 517 nm was measured in a spectrophotometer (Biotek Power Wave XS2/US), and 80% methanol was used for the baseline correction. Scavenging ability was expressed as the inhibition percentage and was calculated by the following equation [18]:

(1) Scavenging ability%=Abscontrol−AbssampleAbscontrol×100,

where Abs_control_ = absorbance of DPPH radical in 80% methanol and Abs_sample_ = absorbance of samples and standards in 80% methanol + DPPH. The antioxidant activity of all samples was expressed as IC_50_, which was defined as the concentration (in *μ*g/mL) of samples required to inhibit the formation of DPPH radicals by 50%. IC_50_ values were calculated by Probit analysis [19]. All assays were performed in triplicate.

### 2.6. Determination of Cytotoxicity by the MTT Assay

The cytotoxic potential of the extract and fractions was evaluated using the MTT assay [20]. LLC-MK2 (Rhesus monkey kidney) cells were maintained at 37°C, under 5% CO_2_, in DMEM supplemented with 5% FBS, 50 U/mL penicillin, and 50 *μ*g/mL streptomycin in a 96-well microplate, until they reach 95% confluence. After 72 h exposure to dosages from 400 to 3.125 *μ*g/mL, 20 *μ*L (2 mg/mL) of phosphate buffered saline (PBS) was added to each well and the plate was incubated at 37°C for 3 h. The medium was removed and 130 *μ*L DMSO was added. After incubation at 37°C for 10 min, the absorbance at 540 nm was measured in a spectrophotometer (Biotek Power Wave XS2, USA) to determine the concentration that killed 50% of the cells (CC_50_). The cytotoxicity was calculated after comparing with the control (treated with 0.1% DMSO).

### 2.7. Culture and Maintenance of the Bacterial Isolates

Eight bacterial strains, which included Gram-positive* Bacillus cereus* ATCC 11778,* Enterococcus faecalis* ATCC 19433,* Listeria monocytogenes* ATCC 15315,* Staphylococcus aureus* ATCC 29213, and* Streptococcus mutans* ATCC25175 and Gram-negative* Escherichia coli* EHEC ATCC 43895,* Klebsiella pneumoniae* ATCC 27736, and* Pseudomonas aeruginosa* ATCC 25853, were used in the biological assays. All bacterial strains were stored at −80°C.

Suspensions from the cultures of the bacteria were prepared in accordance with the guidelines in the CLSI M7-A7 document [21] to obtain a final suitable inoculum of 1.5 × 10^6^ UFC/mL.

### 2.8. Determination of the Minimal Inhibitory Concentration

The minimal inhibitory concentration (MIC) values were obtained by broth microdilution testing performed in accordance with the guidelines in the CLSI M7-A7 document [21], with modifications. Streptomycin and penicillin were included as positive control. The stock solution was prepared in water. MH medium without samples or solvents was used as a control for growth and sterility.

The EE and fractions were dissolved in sterile 20% dimethylsulfoxide (DMSO). Later, serial dilutions were made with MH, maintaining a constant volume of 1000 *μ*L in each tube. In this way, the samples were tested at eight concentrations that varied from 15.62 to 2000 *μ*g/mL and the concentrations of the positive control varied from 7.81 to 1000 *μ*g/mL. An inoculum of 125 *μ*L of cell culture was added to 25 *μ*L of each concentration of samples in MH in 96-well plates. DMSO at 2% (v/v) was used as a control for toxicity.

After inoculation of bacteria, the plates were incubated at 37°C for 24 h. The MIC values were expressed in *μ*g/mL and correspond to the lowest concentrations that inhibited 80% of bacterial growth, by measuring the absorbance at 540 nm (Biotek Power Wave XS2, USA). All assays were performed in triplicate and repeated at least once.

### 2.9. Determination of Minimal Bactericidal Concentration

The minimum bactericidal concentration (MBC) of the EE and fractions was determined by streaking 25 *μ*L from each well that showed inhibition of 80% of bacterial growth onto an agar plate and counting by a Pour-Plate Method [22]. After incubation at 37°C for 24 h, colonies were counted. The MBC was determined as the lowest sample concentration at which less than 0.1% of the initial inoculum (1.5 × 10^6^ UFC/mL) was able to grow. All assays were performed in triplicate and repeated at least once.

### 2.10. Evaluation of the* In Vivo* Anti-Inflammatory Activity

Carrageenan induces paw edema biphasically: the initial phase (0 to 2.5 h) is characterized by the release of histamine, serotonin, and bradykinins, and the later phase begins due to the overproduction of prostaglandins such as PGE_2_ in paw [23].

The volume of the Swiss mice paws was measured with a plethysmometer (Insight, Brazil), according to a method reported previously [24]. The basal volume of the left hind paw was determined before the administration of any drugs. Vehicle (DMSO 5% in saline, 10 mL/kg, control group), ethanol extract, hexane, dichloromethane, ethyl acetate, and hydroethanol fractions (all at doses 75 and 150 mg/kg) or indomethacin 10 mg/kg (*n* = 6 per group) was intraperitoneally administered 30 minutes before the intraplantar (i.pl.) injection of carrageenan (400 *μ*g/paw, 30 *μ*L). The paw volume was measured 1, 2, 4, and 6 h after the injection of the inflammatory stimulus. The difference between the paw edema after and before (basal volume) carrageenan injection was taken as the volume of edema and was determined for each mouse. The percentage of edema inhibition in treated animals was calculated in comparison to the control group (vehicle).

This experiment was in agreement with the Ethical Principles in Animal Experimentation adopted by the Ethics Committee in Animal Experimentation of Universidade Federal de São João Del Rei, Brazil (CEUA/UFSJ) (Protocol 028/2013).

### 2.11. Statistical Analysis

Student's *t*-test was utilized to evaluate the statistical difference between the control group and the group exposed to the EE and fractions of* S. lycocarpum.* Data of paw edema are presented as means ± standard error of the mean (S.E.M.) of measurements. For statistical analysis, the data were analyzed by a one-way analysis of variance (ANOVA), followed by the Bonferroni's post hoc test for multiple comparisons. A *p* value 0.05 was considered statistically significant. The analyses were performed using the GraphPad Prism 5.0 software (San Diego, CA, USA).

## 3. Results and Discussion

### 3.1. Antioxidant Activity, Total Flavonoids Content, and HPLC Analysis

The scavenging effects on the DPPH radical of the ethanol extract and fractions obtained from the leaves of* S. lycocarpum* and IC_50_ values are shown in Table 1. Ethanol extract, fractions, and positive controls (ascorbic acid (AA) and 2,6-di-tert-butyl-4-methylphenol (BHT)) were capable of scavenging DPPH radicals in a concentration-dependent manner. The samples exhibited activities greater than the positive control BHT, at concentrations of 1 and 10 *μ*g/mL. As can be seen from the IC_50_ values (Table 1), dichloromethane (DCM), ethyl acetate (Ac), and hydroethanol (HE) fractions were the most potent antioxidants, with an IC_50_ of 4.29, 1.82, and 3.46 *μ*g/mL, respectively, having an activity better than BHT, a commercial antioxidant (IC_50_ = 16.36 *μ*g/mL). The ethanol extract (EE) and hexane fraction (Hex) showed low antioxidant potential, with high IC_50_ values. The Ac fraction can be considered the best antioxidant among all of the extracts under investigation. The EE and fractions were less active than AA, with an IC_50_ of 1.62 *μ*g/mL.

The IC_50_ values found for the EE and fractions of* S. lycocarpum* on the DPPH radical were statistically significant when compared with BHT and AA. The *p* value for comparison of the EE and fractions of* S. lycocarpum* with BHT was 0.001. The *p* value for the comparison of the EE and fractions of* S. lycocarpum* with AA was 0.001, except for the Ac fraction, with a *p* value > 0.05.

In a previous study on the ripe fruits of* S. lycocarpum*, the ethanol extracts and fractions exhibited antioxidant activity by the DPPH method, with IC_50_ values estimated to be between 2.96 and 172.00 *μ*g/mL [13]. The dichloromethane, ethyl acetate, and hydroethanol fractions from leaves of* S. lycocarpum* presented lower IC_50_ values than the similar fractions of ripe fruits of* S. lycocarpum*, exhibiting a greater antioxidant activity.

The dichloromethane, ethyl acetate, and hydroethanol fractions had the highest total flavonoid content: 168.00, 184.00, and 105.00 *μ*g equivalents to rutin/mg of fraction, respectively. The total flavonoids contents of ethanol extract and hexane fraction were estimated to be 8.40 and 3.40 *μ*g equivalents to rutin/mg of extract or fraction, respectively (Table 1). Phenolic compounds, such as flavonoids, have very strong antioxidant activity and are much more effective than vitamins C and E in protecting cells from free radical damage [25, 26].

**Figure 1:**
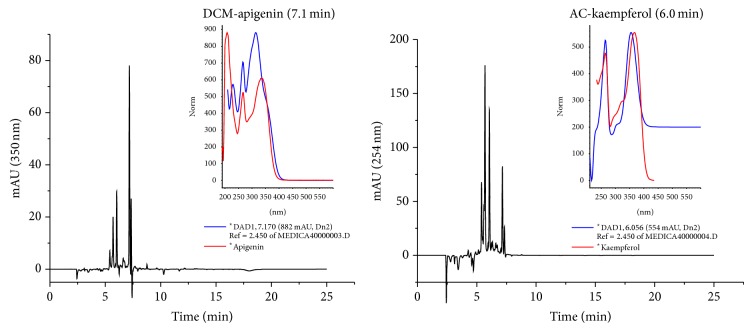
HPLC chromatograms of extract and fractions from* Solanum lycocarpum* by elution with water acidified with chromic acid (0.1% v/v) and acetonitrile, monitored at 350 and 254 nm; dichloromethane fraction (DCM) and ethyl acetate fraction (Ac). Major peaks (DCM) apigenin and (AC) kaempferol derivatives.

The chromatograms of HPLC-DAD of samples of leaves of* S. lycocarpum* revealed the presence of flavonoids. Flavone and flavonol (apigenin and kaempferol) were detected in the dichloromethane and ethyl acetate fractions, respectively (Figure 1). No phenolic compounds were detected in the ethanol extract and hexane and hydroethanol fractions. In the DCM fraction chromatogram, some peaks could be noted. In this chromatogram, a peak (observed between 7.0 and 7.1 min) had similar spectrum with apigenin (264, 298 sh, 311 nm). In the chromatogram obtained with the AC fraction, a spectrum similar to kaempferol derivative (264, 301 sh, 356 nm) was observed at 6.0 min, as the second most pronounced peak (Figure 1). The flavonoids apigenin and kaempferol have been identified in other species of the genus* Solanum*, such as* S. crinitum* and* S. nigrum* [27, 28]. The flavonoids are plant natural compounds and an integral part of the human diet, with increasing evidence that dietary polyphenols are likely candidates for the observed beneficial effects of a diet rich in fruits and vegetables for the prevention of several chronic diseases [29].

### 3.2. Cytotoxic Activity

The cytotoxicity of the EE and fractions from leaves of* S. lycocarpum* was evaluated in the LLC-MK2 cell line by the MTT colorimetric assay. The results showed that the Ac fraction presented less of a cytotoxic effect than the EE and other fractions, with CC_50_ = 9975 *μ*g/mL (Table 1). The DCM fraction showed little cytotoxic activity, with CC_50_ value of 165.70 *μ*g/mL. The EE and Hex and HE fractions showed highest cytotoxicity, with CC_50_ values of 10.20, 13.51, and 25.00 *μ*g/mL, respectively.

In a previous study on the ripe fruits of* S. lycocarpum*, the ethanol extracts and fractions were evaluated in the LLC-MK2 cell line by the MTT assay. The results showed that the Hex fraction presented less of a cytotoxic effect, the EE and HE fraction showed moderated cytotoxicity, and the DCM and Ac fractions showed little cytotoxic activity [13]. Glycoalkaloids from the fruits of* S. lycocarpum* were also evaluated for cytotoxic activity, using LLC-MK2 cells. The results revealed that the samples were effectively nontoxic to the cells [15], corroborating results in this study for the DCM and Ac fractions.

### 3.3. Antibacterial Activities

The antibacterial activities of the EE and fractions from leaves of* S. lycocarpum* were evaluated against eight bacterial strains of clinical interest (Table 2). The fractions presented good antibacterial activity against the Gram-positive bacteria* L. monocytogenes*, with MIC values in the range of 500–2000 *μ*g/mL. The HE fraction was more active against* L. monocytogenes,* with an MIC value of 500 *μ*g/mL. The DCM and HE fractions were also active against* B. cereus,* with an MIC value of 1000 *μ*g/mL. These results are interesting, because both of these bacteria are food-borne pathogens responsible for gastrointestinal infections with high morbidity [30].* B. cereus* is a food-poisoning pathogen frequently diagnosed as the causative agent of gastroenteritis, but it may also cause more severe diseases, such as endophthalmitis and meningitis [30, 31].

The Hex fraction was also active against* S. aureus*,* S. mutans,* and* E. coli*, with MIC values of 2000, 1000, and 1000 *μ*g/mL, respectively. The DCM fraction showed good activity against* S. aureus and K. pneumoniae,* with an MIC of 2000 *μ*g/mL. The Ac fraction was active against* P. aeruginosa* (MIC = 2000 *μ*g/mL). Conversely, the EE extract was active against bacteria tested.

The MBC of the fractions are shown in Table 2. The DCM and HE fractions showed bactericidal activity against* L. monocytogenes*, with MBC values of 2000 and 1000 *μ*g/mL, respectively. The DCM fraction against* L. monocytogenes* showed an MBC result that was equivalent to the MIC result. It can be concluded that this sample exhibited bactericidal and bacteriostatic effects at the same concentration. The MIC and MBC values found for the EE and fractions of* S. lycocarpum* were statistically significant when compared with streptomycin and penicillin, with a *p* value of 0.0001.

The Solanaceae family has revealed its potential as antimicrobial agents.* Solanum* species have also been described as possessing antibacterial properties against strains of medical importance [13, 32–34]. The ethanol extract and fractions from the ripe fruits of* S. lycocarpum* present activity against* B. cereus*,* E. faecalis*,* L. monocytogenes*,* S. aureus*,* S. mutans, S. pyogenes,* and* K. pneumonia,* with MIC values in the range of 31–2000 *μ*g/mL [13]. Acetone and methanol extracts of* Solanum nigrum* leaves exhibited activity against* B. cereus, S. epidermidis, S. aureus, Micrococcus kristinae,* and* P. aeruginosa*, with an MIC of 5 mg/mL [33].* S. tuberosum* showed the highest activity against* S. aureus* and* E. faecalis,* with MIC values of 15.6–31.3 *μ*g/mL [34]. Extracts of the fruits of* Solanum incanum* and* S. nigrum* were shown to be active against* S. aureus*,* B. subtilis*,* E. coli*,* P. aeruginosa*, and* Micrococcus flavus*, with MIC values in the range of 500–1000 *μ*g/mL [32].

### 3.4. *In Vivo* Anti-Inflammatory Activity

The anti-inflammatory activity of the EE and fractions from leaves of* S. lycocarpum* was evaluated by paw edema-induced carrageenan. The ethanol extract, hexane, and dichloromethane fractions did not inhibit paw edema (data not shown).

**Figure 2:**
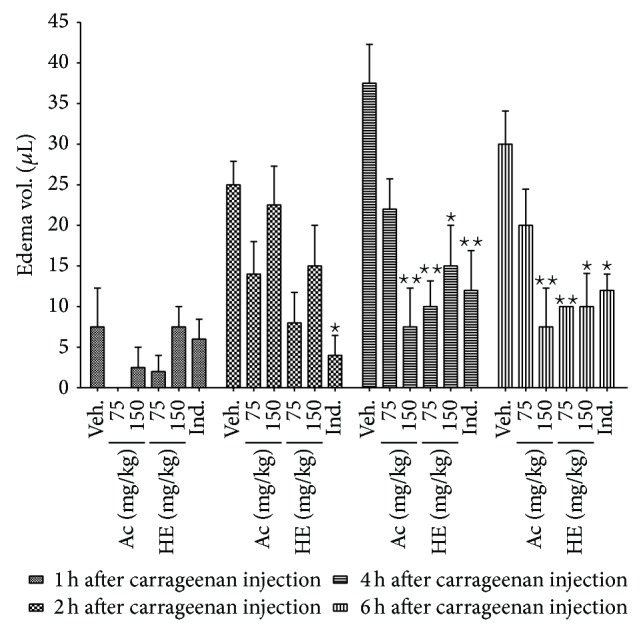
Anti-inflammatory effect of hydroethanol (HE) and ethyl acetate (Ac) fractions from* S. lycocarpum* versus time after inflammatory stimulus on carrageenan-induced paw edema. Paw edema was measured with plethysmometer; results expressed as paw edema in *μ*L (mean ± SEM); treatments: HE and Ac fractions from* S. lycocarpum* (75 and 150 mg/kg), vehicle (Veh. 5% DMSO), or indomethacin (Ind. 10 mg/kg) thirty minutes before intraplantar carrageenan injection (400 *μ*g/paw). *n* = 6 animals/group. ANOVA, Bonferroni's Multiple Comparison Test; ^∗^ *p* < 0.05 and ^∗∗^ *p* < 0.01 in comparison to control group (vehicle).

Both applied doses of HE fraction (75 and 150 mg/kg) and 150 mg/kg of Ac fraction from* S. lycocarpum* leaves exerted a significant anti-inflammatory action in the mice paw edema test (Figure 2). The suppression of local edema formation by the higher dose of HE and Ac fractions was 60% and 80%, respectively, 4 h after injection of carrageenan and 67% and 75%, respectively, 6 h after the inflammatory stimulus. The inhibition of edema observed was pronounced in the later phase of inflammation, which was similar to the effect of nonsteroidal anti-inflammatory drugs such as indomethacin [35], indicating that the antiedematogenic activity is possibly mediated through a cyclooxygenase enzyme inhibitory pathway [36].

The ethyl acetate fraction had better anti-inflammatory and antioxidant activities and the highest total flavonoid content. Phenolic compounds such as flavonoids possess anti-inflammatory activity, which is related to their antioxidant activity [37]. The inflammatory process is a complex response and reactive oxygen species play an important role in the pathogenesis of inflammatory diseases [38]. One of the anti-inflammatory mechanisms of phenols seems to be related to the inhibition of NF-*κ*B transcription and the reduction of the expression of several target genes that are indispensable to the inflammatory process [39–41]. Kaempferol, identified as the constituent of the Ac fraction (Figure 1), could account for the antiedematogenic effect. A previous study demonstrated that the anti-inflammatory property of kaempferol is related to the inhibitory effect on nitric oxide (NO) production and NF-*κ*B mediated luciferase activity [5].

## 4. Conclusion

The DCM, Ac, and HE fractions were the most potent antioxidants, with an activity that was better than BHT, and also have the highest total flavonoid content. In the DCM and Ac fractions, the flavonoids (apigenin and kaempferol, resp.) were detected. The Hex and DCM fractions were more active than the EE and other fractions against the tested bacteria. In addition, we confirm the anti-inflammatory activity of HE (75 and 150 mg/kg) and Ac (150 mg/kg) fractions from* S. lycocarpum* leaves. The Ac fraction also presented a less cytotoxic effect than the EE and other fractions. These activities found in* S. lycocarpum* leaves can be attributed, at least partially, to the presence of phenolic constituents such as flavonoids. These results encourage additional studies to evaluate the possibilities of using the extract and fractions of leaves of* S. lycocarpum* as a potential source of natural antioxidant, antibacterial, and anti-inflammatory compounds in complementary and alternative therapeutics.

## Figures and Tables

**Table 1:** DPPH-scavenging activity, IC_50_ values, total flavonoids, and cytotoxic activity of ethanol extract and fractions from leaves of *Solanum lycocarpum*.

Samples	DPPH scavenging activity					IC_50_ (*μ*g/mL)	TFC	CC_50_ (95% CL)
	1 *μ*g/mL	10 *μ*g/mL	100 *μ*g/mL	250 *μ*g/mL	500 *μ*g/mL			*μ*g/mL
EE	32.03 ± 0.42^ab^	37.16 ± 0.34^ab^	93.69 ± 0.23^ab^	97.42 ± 0.06^ab^	99.38 ± 0.27^a^	36.63 ± 6.06^ab^	8.40 ± 0.74	10.20 ± 1.54
Hex	38.66 ± 0.52^a^	41.82 ± 0.64^ab^	47.29 ± 0.58^ab^	58.28 ± 0.91^ab^	67.02 ± 0.69^ab^	178.14 ± 33.63^ab^	3.40 ± 0.47	13.51 ± 3.72
DCM	33.34 ± 0.24^ab^	66.00 ± 0.17^ab^	71.37 ± 0.62^ab^	83.56 ± 0.83^ab^	99.59 ± 0.19^a^	4.29 ± 0.89^ab^	168.00 ± 2.89	165.70 ± 4.00
Ac	43.48 ± 0.29^ab^	65.73 ± 0.73^ab^	76.65 ± 0.29^ab^	92.43 ± 0.28^ab^	97.10 ± 0.69^ab^	1.82 ± 0.32^a^	184.00 ± 1.73	9975.00 ± 5.41
HE	32.09 ± 1.67^ab^	65.88 ± 0.84^ab^	87.80 ± 0.35^ab^	91.91 ± 0.40^b^	98.41 ± 0.05^ab^	3.46 ± 0.45^ab^	105.00 ± 1.15	25.00 ± 2.01
BHT	18.50 ± 0.24	25.90 ± 0.64	86.00 ± 0.56	91.40 ± 0.28	94.02 ± 0.64	16.36 ± 1.63	—	—
AA	39.10 ± 0.34	82.60 ± 0.26	90.80 ± 0.32	95.08 ± 0.43	99.80 ± 0.58	1.62 ± 0.25	—	—

Ethanol extract (EE), hexane (Hex), dichloromethane (DCM), ethyl acetate (Ac), and hydroethanol (HE) fractions, 2,6-di-tert-butyl-4-methylphenol (BHT) and ascorbic acid (AA).

IC_50_: concentration (in *μ*g/mL) of samples required to inhibit the formation of DPPH radicals by 50%.

TFC: total flavonoids content: results expressed as *μ*g of rutin equivalents/mg of extract or fraction.

CC_50_: concentration that killed 50% of cells; CL: confidence limits.

Each value in the table is the mean ± standard deviation (*n* = 3). ^a^ *p* < 0.05 compared with BHT, ^b^ *p* < 0.05 compared with AA.

**Table 2:** Minimal inhibitory concentration (MIC) and minimal bactericidal concentration (MBC) of ethanol extract and fractions from leaves of *Solanum lycocarpum* against eight bacteria of clinical interest.

Bacteria	Concentration (*μ*g/mL)											
	EE		Hex		DCM		Ac		HE		Streptomycin/penicillin	
	MIC	MBC	MIC	MBC	MIC	MBC	MIC	MBC	MIC	MBC	MIC	MBC
*Bacillus cereus *	>2000	—	>2000	—	1000^∗^	>2000	>2000	—	1000^∗^	>2000	7	1000
*Enterococcus faecalis *	>2000	—	>2000	—	>2000	—	>2000	—	>2000	—	1000	1000
*Listeria monocytogenes *	>2000	—	2000^∗^	>2000	2000^∗^	2000^∗^	>2000	—	500^∗^	1000	125	125
*Staphylococcus aureus *	>2000	—	2000^∗^	>2000	2000^∗^	>2000	>2000	—	>2000	—	7	125
*Streptococcus mutans *	>2000	—	1000^∗^	>2000	>2000	>2000	>2000	—	>2000	—	7	7
*Escherichia coli *	>2000	—	1000^∗^	>2000	>2000	—	>2000	—	>2000	—	15	30
*Klebsiella pneumoniae *	>2000	—	>2000	—	2000^∗^	>2000	>2000	—	>2000	—	15	15
*Pseudomonas aeruginosa *	>2000	—	>2000	—	>2000	—	2000^∗^	>2000	>2000	—	62	125

Ethanol extract (EE), hexane (Hex), dichloromethane (DCM), ethyl acetate (Ac), and hydroethanol (HE) fractions.

^∗^ *p* < 0.05 compared with streptomycin/penicillin.
